# Psychosocial determinants of sustained maternal functional impairment: Longitudinal findings from a pregnancy-birth cohort study in rural Pakistan

**DOI:** 10.1371/journal.pone.0225163

**Published:** 2019-11-19

**Authors:** Ashley Hagaman, John A. Gallis, Sonia Bhalotra, Victoria Baranov, Elizabeth L. Turner, Siham Sikander, Joanna Maselko

**Affiliations:** 1 Department of Social Behavioral Sciences, Yale School of Public Health, New Haven, Connecticut, United States of America; 2 Center for Methods in Implementation and Prevention Sciences, Yale University, New Haven, Connecticut, United States of America; 3 Duke Global Health Institute, Duke University, Durham, North Carolina, United States of America; 4 Department of Biostatistics and Bioinformatics, Duke University, Durham, North Carolina, United States of America; 5 Department of Economics, University of Essex, Essex, England, United Kingdom; 6 Department of Economics, The University of Melbourne, Melbourne, Australia; 7 Maternal and Neonatal Child Health Department, Health Services Academy, Islamabad, Pakistan; 8 Human Development Research Foundation, Islamabad, Pakistan; 9 Department of Epidemiology, Gillings School of Global Public Health, University of North Carolina at Chapel Hill, Chapel Hill, North Carolina, United States of America; King Fahd University of Petroleum & Minerals, SAUDI ARABIA

## Abstract

Function is an important marker of health throughout the life course, however, in low-and-middle-income-countries, little is known about the burden of functional impairment as women transition from pregnancy to the first year post-partum. Leveraging longitudinal data from 960 women participating in the Share Child Cohort in Pakistan, this study sought to (1) characterize functional trajectories over time among women in their perinatal period and (2) assess predictors of chronic poor functioning following childbirth. We used a group-based trajectory modeling approach to examine maternal patterns of function from the third trimester of pregnancy through 12 months post-partum. Three trajectory groups were found: persistently well-functioning (51% of women), poor functioning with recovery (39% of women), and chronically poor functioning (10% of women). When compared to mothers in the highest functioning group, psychosocial characteristics (e.g., depression, stress, and serious life events) were significantly associated with sustained poor functioning one-year following child-birth. Mothers living in nuclear households were more likely to experience chronic poor functioning. Higher education independently predicted maternal function recovery, even when controlling for psychosocial characteristics. Education, above and beyond socio-economic assets, appears to play an important protective role in maternal functional trajectories following childbirth. Public health implications related to maternal function and perinatal mental health are discussed.

## Introduction

Function and related impairment are important markers of health throughout the life course. Function, an individual’s ability to perform their daily activities, is an integral dimension of an individual’s overall quality of life and a valuable domain when considering the severity, prognosis, and treatment approach to various health issues [1]. Assessing functional impairment in the clinic or community may provide more insight than disease diagnosis alone, giving care providers and patients vital information relating to service needs, amenable clinical and social interventions, and monitoring outcomes. The perinatal period (between pregnancy and one year following childbirth) is a window of high morbidity and mortality for both the mother and infant, and remains a global health priority [2, 3]. Motherhood, in addition to conferring a new identity and social role, maternity also offers women important social mobility and opportunities for personal fulfillment, allowing women to achieve new dimensions of life satisfaction [4, 5]. Particularly in low-and-middle-income-countries (LMIC), the perinatal period is critically linked to future health, social, and economic outcomes of both mother and her child. Further understanding patterns and fluctuations of maternal function throughout the perinatal period can shed important insights into how varying morbidities and environmental stressors manifest in a mother’s ability to engage in meaningful aspects of her life [6]. Additionally, research examining maternal functional recovery can provide insights into policies and implications for mothers returning to work and daily tasks, particularly in resource-strained settings where poverty has compounding effects.

Despite the importance of function and quality of life among mothers, and the significant milestone of childbirth, there is a paucity of evidence documenting such trajectories from pregnancy through one-year postpartum. Global efforts to characterize the burden of disability as it relates to disease and injury is well underway [7]. This approach, however, focuses on disabilities related to specific disease and injury states. The disability burden is well established in high-income countries and aging populations [8]. Studies in high-income countries found that low quality of life during the perinatal period increases risks for the fetus (pre-term birth), the infant (low birth weight and poor development outcomes), and maternal physical and mental health [9, 10]. However, little is known about fluctuations in function over time (e.g., functional trajectories), particularly throughout the perinatal period for women in low-income settings [11].

Longitudinal links between function, disability and depression have also been made, however, this literature is limited to older populations and higher income settings [12]. Understanding the impacts of psychosocial factors on function throughout the perinatal period may be important, particularly because low function significantly impacts quality of life and care-seeking. Psychosocial factors broadly refer to the combination of the social environment and individual-level psychological domains, such as social support or psychological well-being, that shape other domains of health [13]. This is particularly important to close gaps in antenatal and postnatal care. In recent years, investigations into assessments, burdens, risk-factors, and treatments for maternal mental disorders have established a growing evidence base in LMIC [14–16]. Perinatal depression may affect as many as 40% of women in some LMIC contexts and significantly increases morbidity and disability among recent mothers [16, 17]. Research during pregnancy has highlighted how depression and social support exacerbates the expected physical and mental tolls pregnancy and childbirth inflict. Depressed pregnant women report worse physical, mental, and social functioning compared to non-depressed [18–20]. Additionally, women with less social support experience higher levels of postpartum depression [17, 21, 22]. Similarly, postpartum depression significantly impacts a new mother’s quality of life and functional capability, but less is known about how psychosocial factors (such as stress, depression, and social support) affect functional recovery beyond the immediate postpartum period [9]. While maternal depression studies often measure daily functioning, little detail is typically shared regarding the fluctuations in functional impairment over time for women during the perinatal period. Such information can help disentangle the roles common mental disorders and other social indicators play in maternal function, and overall maternal morbidity, over time. To this end, this study examines how functional impairment fluctuates throughout the perinatal period, and how functional trajectories relate to psycho, social, and economic factors in a population-based sample of perinatal women in peri-urban Pakistan. This study aims to fill the aforementioned gaps by (1) characterizing functional trajectories over time among women in their perinatal period and (2) assessing predictors of chronic poor functioning (e.g., sustained for up to one year) following childbirth.

## Materials and methods

### Sample

We used data collected as part of the cohort study following participants of the Thinking Health Peer Delivered Plus (THPP+) program, a cluster randomized controlled trial of a perinatal depression intervention in rural Pakistan. All pregnant women in the study area were identified and screened for depression. All positively screened depressed women were invited for participation along with an equal number of non-depressed women. Depressed women in the intervention areas began the program and all women were interviewed in their last trimester of pregnancy (baseline) and multiple times during the follow-up postnatal period. Complete details of the study’s protocol, trial results, and cohort characteristics can be found elsewhere [23–25]. For the current analyses, we used 15 months of longitudinal data collected from 1,154 women who were interviewed at baseline, 3, 6, and 12 months post-partum. To make the sample representative of the local population, we weighted non-depressed women (PHQ-9 < 10) to account for their sub-sampling during study sample recruitment (approximately 1 in 3 non-depressed women screened was included in the study). These weights were specific to the cluster. All non-depressed women in a cluster were weighted by the inverse proportion of non-depressed women screened for depression in that cluster who were subsequently enrolled in the study [24, 26]. Since all depressed women were invited to participate in the study, all received a weight of 1. Cluster-specific weights were applied for the regressions in order to generalize the results to the population from which the sample was drawn. In order to create reliable trajectories, women who missed two or more of the four interviews were excluded, resulting in a total sample of 960. Sensitivity analyses indicated no evidence that the included women are not representative of the entire enrolled sample. The amount and type of missing data across assessments in both the retained and dropped samples can be found in S1 and S2 Tables.

### Measures

Function was assessed using the 12-item World Health Organization Disability Assessment Schedule 2.0 (WHODAS 2.0), an instrument with high validity and internal consistency across cultures and contexts, particularly in LMIC [27]. The WHODAS measures two distinct factors related to daily functioning: activity (e.g. difficulty standing, walking, doing household work) and social participation (e.g. learning a new task, maintaining a friendship) and has an overall range of 0 to 48. The WHODAS 2.0 is described as a “generic assessment instrument for health and disability used across all diseases” and is applicable in both clinical and population settings. The WHODAS has been used to assess daily functioning in several countries around the world [28–31]. We interpret our findings in the context of general daily functioning and do not make attempts to characterize specific disability levels or profiles.

Other variables included in the present analysis were measured at baseline, unless noted otherwise. Depression was assessed by the PHQ-9 [32]. Perceived stress was assessed by the 10-item Cohen Perceived Stress Scale [33]. Social support was measured using the multidimensional scale of perceived social support [34]. Social economic status was assessed using a standard asset inventory. The asset index was calculated using a polychoric correlation principal components analysis [26]. A modified checklist from the Life Events and Difficulties Schedule (such as a close relative dying or significant marital problems) was adapted for the study setting and culture and a 10-item index subsequently calculated that totaled the number of significant life events endorsed [35]. We assessed longstanding chronic illness or disability through self-report. We adjusted models for Intimate partner violence (IPV) in the past year as well as any history of obstetric outcomes. IPV was assessed at baseline using the World Health Organization Violence Against Women Instrument [36]. A three-level variable was constructed to indicate if a woman experienced any IPV, no IPV, or did not respond. Any history of obstetric outcomes (e.g., miscarriages and/or stillbirths) and deaths of children prior to the index pregnancy were also elicited at baseline. A composite variable was created to indicate if any miscarriage, stillbirth, or death of a child under the age of five occurred prior to the birth of the index child. Other socio-demographic characteristics included maternal education, age of marriage, number of living children, gender of the index infant, and household structure. Education was collapsed into four categories: no education, primary (equivalent to completing grades 1 through 5), middle (equivalent to completing grades 6 through 8), and secondary or more (completing more than 9 grades). Number of living children was categorized into three groups including nulliparous mothers where her current pregnancy was her first, women with one to three children, and women with four or more children not including her current pregnancy. Household structure included either living in a nuclear household, in a joint/extended household (e.g., multiple generations sharing one kitchen), or in a multiple household compound (e.g., multiple generations have separate kitchens, but live in a shared compound). All items and instruments were culturally adapted, translated, and back-translated, under-going an iterative process until face validity was established.

### Statistical analysis

Trajectories of function were determined using a group-based mixture method that identifies distinct homogenous clusters of similar progressions of outcomes within a population over time [36]. This method has been used in population research to assess patterns of various health outcomes throughout the life course including depression, disability, and risky sexual behavior [12, 37, 38]. The procedure simultaneously fits a censored normal finite mixture model of the WHODAS 2.0 as a function of time and a latent class model using the multinomial logistic regression of the trajectory classification. Following selection and reporting procedures outlined by Nagin et al (2005; 2010), we used a dual stage selection process, guided by our research questions and utility of the final model, to identify appropriate group trajectories [39]. First, we determined the best fitting number of trajectory groups by assessing the Bayesian information criterion (BIC) and Akaike information criterion (AIC) for various number of groups. Groups were sequentially added to the model until fit statistics were optimized. Second, we assessed the posterior probabilities of group membership and required that that average posterior probability reached 0.70 or higher to be a distinct classification group. Additionally, we required at least 5% of the sample to be present in a particular group. We determined each trajectory’s trend by systematically testing all combinations for the best fitting line for each group (linear, quadratic, or cubic). Similar methods have been applied in psychosocial and disability research cohorts [12, 38].

To assess what participant characteristics significantly affected the probability of group membership, we performed simple multinomial logistic regressions, applying sampling weights generalize the regression results to the population from which the sample was drawn, using predictors of participant social, demographic, and psychometric characteristics with the low-level disability group as the outcome reference group. First, unadjusted bivariate associations between each characteristic and probability of group membership were tested (reported in S3 Table). Second, we sequentially fit multinomial logistic regression models to determine factors associated with membership to poorer functioning groups. Model 1 adjusted for demographic and socioeconomic factors including age, number of living children, socioeconomic assets (SES), maternal education, and household structure. Next, we further adjusted Model 2 for illness (baseline report of chronic illness and baseline depression symptoms) in addition to covariates included in Model 1. Third, we created a fully adjusted Model 3, adding psychosocial factors including social support, significant life events, and perceived stress, as well as controlling for experiencing IPV at baseline, the index infant’s gender, and any history of miscarriage, stillbirth, or previous child death. We report the odds ratio of group placement using the odds of belonging to the poorer function groups relative to the high function group for the variables of interest.

## Results

**Fig 1:**
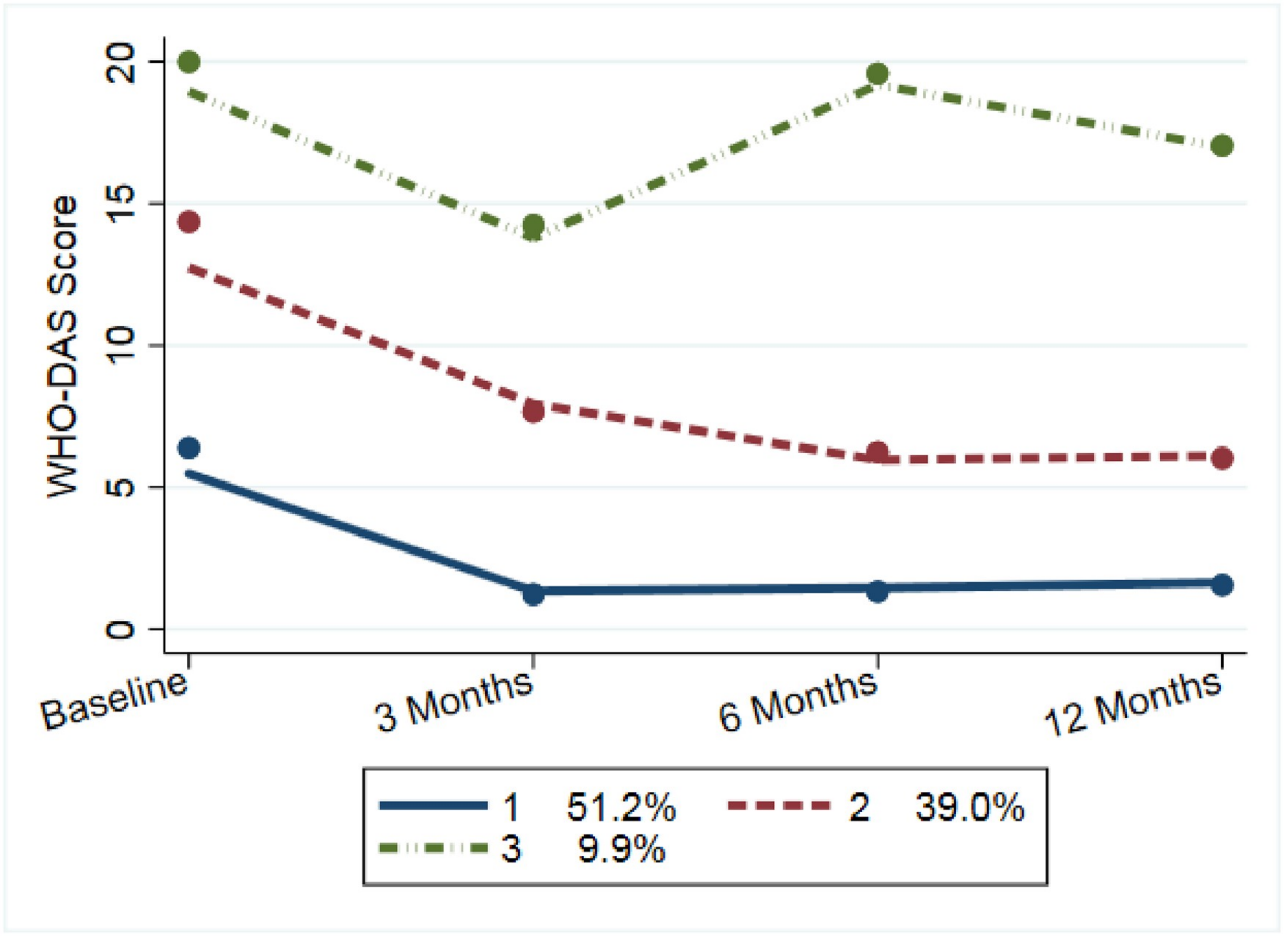
Perinatal function trajectory classifications among mothers (n = 960).

Sample characteristics are reported in Table 1 and data availability in Table 2. About one-third of women had low or no education, one-third were in their first pregnancy, nearly 80% lived in extended or multi-family households, and 12.5% reported having a long-standing illness or disability at baseline. We found that three trajectory groups best fit the data and that cubic, quadratic, and cubic patterns were the best fitting lines for group 1, 2, and 3 respectively. To explore the extent to which individuals were appropriately classified to a given trajectory, we report the mean posterior probabilities of placement into each group in Table 3. Results indicate good trajectory classification, as the probabilities for a set of profiles are closer to one in a specific trajectory class while closer to zero for other trajectories. For example, the mean probability for profiles assigned to Group 1 was 0.86, while the means for Groups two and three were 0.15 and 0.00 respectively. Function trajectories and the proportion of our sample assigned to each group can be found in Fig 1. The three trajectories were defined by: (1) persistently well-functioning (51% of women), (2) poor functioning with modest recovery (39.0% of women), and (3) chronically poor functioning (9.9% of women). The majority of women experienced initial high levels of functional impairment, which is expected as pregnancy itself can be disabling as a woman nears childbirth. There are two distinct groups, however, that either experience very little disablement both before and after pregnancy, and another group that experiences chronic higher levels without recovery. An increasingly disabled category did not emerge. The low and higher-level disability groups followed cubic trajectories while the recovering group followed a quadratic pattern (Fig 1).

**Table 1:** Demographics table by trajectory group.

	Group 1 (N = 509)	Group 2 (N = 366)	Group 3 (N = 85)	Total (N = 960)
**Age in years**				
Mean (SD)	26.59 (4.42)	26.87 (4.57)	26.56 (4.46)	26.70 (4.48)
Median (Q1, Q3)	26.0 (23.0, 30.0)	26.0 (24.0, 30.0)	26.0 (24.0, 30.0)	26.0 (24.0, 30.0)
% Missing (Min, Max)	0.0% (18.0, 45.0)	0.0% (18.0, 40.0)	0.0% (18.0, 37.0)	0.0% (18.0, 45.0)
**SES Asset Index**				
Mean (SD)	0.32 (1.50)	-0.18 (1.66)	-0.75 (1.68)	0.03 (1.61)
Median (Q1, Q3)	0.6 (-0.4, 1.4)	0.1 (-1.2, 1.0)	-0.6 (-2.0, 0.6)	0.4 (-0.9, 1.2)
% Missing (Min, Max)	0.0% (-4.7, 2.8)	0.0% (-4.5, 2.8)	0.0% (-5.0, 2.6)	0.0% (-5.0, 2.8)
**Grades woman has passed, categorized**				
None (0)	52 (10.2%)	65 (17.8%)	18 (21.2%)	135 (14.1%)
Primary (1–5)	86 (16.9%)	74 (20.2%)	31 (36.5%)	191 (19.9%)
Middle (6–8)	99 (19.4%)	65 (17.8%)	16 (18.8%)	180 (18.8%)
Secondary or more (9–12+)	272 (53.4%)	162 (44.3%)	20 (23.5%)	454 (47.3%)
**Number of living children**				
First pregnancy	170 (33.4%)	94 (25.7%)	16 (18.8%)	280 (29.2%)
1 to 3	307 (60.3%)	233 (63.7%)	56 (65.9%)	596 (62.1%)
4	32 (6.3%)	39 (10.7%)	13 (15.3%)	84 (8.8%)
**Household structure**				
Nuclear	89 (17.5%)	92 (25.1%)	27 (31.8%)	208 (21.7%)
Joint/Multiple households	420 (82.5%)	274 (74.9%)	58 (68.27%)	752 (78.3%)
Joint/extended	363 (71.3%)	233 (63.7%)	40 (47.1%)	636 (66.3%)
Multiple households	57 (11.2%)	41 (11.2%)	18 (21.2%)	116 (12.1%)
**Longstanding illness, disability, or infirmity**				
No	459 (90.2%)	295 (80.6%)	67 (78.8%)	821(85.5%)
Yes	50 (9.8%)	71 (19.4%)	18 (21.2%)	139 (14.5%)

SES: socioeconomic status

**Table 2:** Function assessment (WHODAS) availability.

	Total (N = 960)
**Number of data points**	
Data at all time points	783 (81.6%)
Missing data at 12 months	41 (4.3%)
Missing data at 6 months	43 (4.5%)
Missing data at 3 months	93 (9.7%)

WHODAS: World Health Organization Disability Assessment Schedule

**Table 3:** Group membership probabilities.

	Group 1 (N = 509)	Group 2 (N = 366)	Group 3 (N = 85)
**Probability of membership in group 1**			
Mean (SD)	**0.86** (0.14)	0.15 (0.14)	0.00 (0.00)
**Probability of membership in group 2**			
Mean (SD)	0.14 (0.14)	**0.78** (0.13)	0.18 (0.16)
**Probability of membership in group 3**			
Mean (SD)	0.00 (0.00)	0.07 (0.11)	**0.82** (0.16)

We next examined the correlates of belonging to a given trajectory. Results for sequentially adjusted regression models can be found in Table 4 (unadjusted bivariate comparisons can be found in S3 Table).

**Table 4:** Predictors of group trajectory membership (reference group 1, persistent high functioning (n = 509)).

	Model 1				Model 2				Model 3			
	Moderate Recovery, Group 2 (n = 366)		Chronic poor functioning, Group 3 (n = 85)		Moderate Recovery, Group 2 (n = 366)		Chronic poor functioning, Group 3 (n = 85)		Moderate Recovery, Group 2 (n = 366)		Chronic poor functioning, Group 3 (n = 85)	
	aOR	CI	aOR	CI	aOR	CI	aOR	CI	aOR	CI	aOR	CI
**Demographic and Socioeconomic**												
Maternal Age at baseline	0.96	0.91–1.01	0.92*	0.85–0.99	0.97	0.92–1.02	0.93	0.86–1.01	0.97	0.92–1.02	0.94	0.86–1.02
Number of Living Children (nulliparous is comparison)												
1 to 3	1.22	0.81–1.82	1.06	0.43–2.63	1.19	0.80–1.78	0.99	0.41–2.41	1.15	0.77–1.73	0.91	0.35–2.39
4 or more	1.56*	1.04–2.34	1.61	0.78–3.33	1.35	0.92–1.99	1.11	0.53–2.35	1.29	0.84–1.98	0.96	0.42–2.22
SES Asset Index Score	0.85	0.68–1.06	0.9	0.65–1.24	0.84	0.66–1.07	0.94	0.66–1.35	0.91	0.71–1.17	1.04	0.74–1.48
Maternal Education (none is comparison)												
Primary (1–5)	0.71	0.41–1.22	0.84	0.43–1.61	0.72	0.40–1.32	0.88	0.42–1.83	0.69	0.36–1.29	0.83	0.37–1.84
Middle (6–8)	0.63	0.35–1.15	0.42	0.16–1.07	0.64	0.34–1.21	0.38	0.14–1.01	0.65	0.33–1.28	0.35*	0.13–0.95
Secondary or more (9–12+)	0.72	0.38–1.28	0.29*	0.10–0.88	0.76	0.39–1.49	0.29*	0.09–0.92	0.74	0.36–1.55	0.29*	0.09–0.94
Household structure (joint is comparison)												
Multiple households	0.82	0.47–1.43	1.77	0.95–3.27	0.81	0.47–1.40	2.01*	1.06–3.81	0.75	0.43–1.32	1.67	0.88–3.17
Nuclear household	1.59*	1.01–2.48	2.77*	1.22–6.29	1.63*	1.03–2.58	2.94*	1.29–6.70	1.70*	1.06–2.71	2.89*	1.29–6.38
**Illness**												
Chronic illness or disability at baseline					1.85*	1.05–3.27	2.22*	1.11–4.43	1.63	0.92–2.88	1.73	0.86–3.49
Depression (PHQ-9)					1.17***	1.09–3.50	1.36***	1.26–1.47	1.12**	1.04–1.20	1.29***	1.18–1.41
**Psychosocial Factors**												
Social Support (MSPSS)									0.97	0.83–1.15	1.02	0.81–1.28
Life events									1.11**	1.03–1.20	1.23***	1.12–1.35
Perceived Stress (PSS)									1.04**	1.02–1.07	1.07*	1.01–1.12

Sampling weights applied in all models. Model 1: Adjusted for age, number of living children categorized, socioeconomic assets, maternal education, and household structure; Model 2: Adjusted for covariates in model 1 and chronic illness/disability at baseline and depression score at baseline; Model 3: Adjusted for all covariates in model 2 and psychosocial factors including perceived stress, life events, and social support as well as any IPV in past 12 months at baseline, index infant gender, and any history of stillbirth, miscarriage, or child death before 5 years.

*p-value <0.05;

** p-value <0.01;

***p-value<0.001

Demographic and socioeconomic factors: Living in a nuclear household significantly increased the odds of being selected into the moderate recovery group (OR: 1.59, CI: 1.01–2.48) compared to the persistently well-functioning group in the partially adjusted model (Model 1). In Model 3, which adjusts for all covariates, compared to mothers living in a joint household, living in a nuclear household increased the odds of moderate recovery by almost 70 percent (OR:1.70, CI: 1.06–2.71). The effect of living in a nuclear household increased the odds of poor functioning by three-fold (OR: 2.89, CI: 1.29–6.38) in the fully adjusted model. Education played a significant protective role against sustained poor function in the perinatal period. Mothers with secondary or more education were significantly less likely to be placed in the poorest functioning trajectory, and this effect is enhanced for those with the most education (e.g., secondary or more) in the fully adjusted model (OR: 0.29, CI: 0.09–0.94). Having four or more children significantly increased the odds of placing in the moderate recovery group (OR: 1.56, CI: 1.04–2.34), but did not remain significant for either group placement in adjusted Models 2 or 3. Maternal age and socioeconomic assets were not significantly associated with group placement in either of the poorer functioning groups.

Illness factors: In the fully adjusted model, higher depression symptoms at baseline remained significantly associated with increased odds of placement in both poorer functioning groups (OR:1.12, CI: 1.04–1.20 for the moderate functioning group and OR:1.29, CI: 1.18–1.41 for the poor functioning group). Existing chronic illness or disability at baseline did not remain associated with placement in either group after adjusting for psychosocial factors.

Psychosocial factors: For every one unit increase on the perceived stress scale and for every additional significant life event endorsed, mothers had 7% (CI: 1.02–1.12) and 23% (CI: 1.12–1.35) increased odds respectively of sustained poor functioning (group 3) when adjusting for all other sociodemographic and illness-related factors. Social support was not associated with group placement. See Table 4 for complete results for partially adjusted and fully adjusted models.

## Discussion

To our knowledge, this is the first characterization of longitudinal function trajectories and psycho-social-demographic correlates among a perinatal population in LMIC. Depression symptom severity and higher perceived stress during pregnancy independently predicted sustained functional impairment and less likelihood of functional recovery in the year following childbirth. This finding follows previous maternal depression studies in LMIC [15, 17, 20, 40], and points to the importance of screening and treating depression during pregnancy and continuing to follow mothers postnatally. This has additional implications for child health as fully functioning mothers are critical in the first year of the child’s life [41]. Our findings suggest that decreasing depression and stress before childbirth may be one strategy for closing the functional impairment gap (and increasing maternal capability to return to regular social and work activities) that persists one year postpartum. Further explorations of potential economic and social consequences of this gap may also provide more insights into the benefits of providing antenatal mental health care and support to at-risk populations.

We also found multiple social factors that independently predicted better functioning throughout the perinatal period, including not living in a nuclear household and having more education. It may be that the burden of household responsibilities are less offset in single family living structures, whereas joint households may be more likely to share tasks surrounding childcare, cooking, and cleaning. Higher perceived social support and economic assets were significantly associated with both high functioning and recovery in the partial, but not fully adjusted models. Living in a multi-family household then, may afford added benefits beyond social support and economic resources. The MSPSS measures support in relation to family, friends, and spouse, but still may not be specific enough to the source of support needed. Previous ethnographic work in LMIC found the importance of parental support, not spousal or friendship, buffered against maternal depression [42]. In patrilocal LMIC settings, women typically move into the husband’s household so that she may no longer have access to the support of her own parents and previous social network. Prior research highlights the importance of household structure and maternal depression particularly in relation to spousal cohabitation [43, 44], but not larger family units. Future research examining specific household members’ roles and aspects of a smaller household on maternal functional impairment is needed, particularly in LMIC.

In our analysis, education had the largest independent effect on functional trajectory group prediction than any other correlate. Higher education appeared to bolster resilience and significantly increase likelihood of recovery, above and beyond an asset based measure of SES and other psychosocial indicators. There is rich sociological work supporting that educational attainment and the related abilities individuals foster as a result, drive social status and health outcomes [45–47]. This may be what we are finding in our perinatal population where education provides productive abilities beyond what other SES domains provide. Mirowsky and Ross posit that the education provides valuable human capital (e.g. habits, potential for self-direction, and increased personal control over life events) that can buffer against poor health and increase well-being [45]. In contexts of chronic poverty, education may be one particularly important asset for functional recovery in the perinatal period. Additionally, this finding may be useful when targeting interventions and follow ups as women with less or no education may be additional support. Further investigation into these pathways is needed.

### Limitations

The study has several limitations to consider. Functional impairment was measured with one instrument and may lack important multidimensionality. Future research using validated forms of health related quality of life and activities of daily living scales may provide more insights. We did not analyze processes involved in creating the trajectories, rather, our analysis uses baseline indicators of social support, socio-economic status, and psychosocial predictors. Although we account for the gender of the index child, the composition of gender and age among all children in a family plays complex roles in the psychosocial milieu, particularly in South Asian contexts. Thus, the current analysis is unable to address this. Future work including multiple longitudinal measures of these time-varying correlates is a focus of the team’s ongoing research. While our findings are representative of the local population of perinatal women, generalizations beyond Pakistan are not possible.

## Conclusion

Our findings suggest that most mothers in our Pakistani sample experience moderate functional impairment during pregnancy followed by complete or moderate recovery within one year of childbirth. However, ten percent of mothers experience chronic functional impairment that persists well after the birth of their child. Worse psychosocial characteristics during pregnancy, including depression, perceived stress, and significant life events, significantly increased the odds of placement in the poorest functioning group. Additionally, education and living in a joint household (but not increased social support) appeared to have distinct protective effects, buffering against chronic poor functioning in the postnatal period. Attending to the psychosocial needs of perinatal populations may have far reaching effects on the quality of life among mothers. Targeting mothers living in nuclear households and creating opportunity for women to maximize their education may also be important to optimize maternal function throughout her reproductive years.

## Supporting information

S1 Table Baseline demographics by inclusion and exclusion. (DOCX)

S2 Table Baseline demographics by all time points available versus three time points. (DOCX)

S3 Table Predictors of group trajectory membership (reference group 1, persistent high functioning (n = 509)). (DOCX)

S1 Dataset Bachpan dataset. (DTA)
