# Supplementary material for: Psychosocial determinants of sustained maternal functional impairment: Longitudinal findings from a pregnancy-birth cohort study in rural Pakistan
Source: PLoS One. 2019 Nov 19;14(11):e0225163. doi: 10.1371/journal.pone.0225163 (PMC6863521; doi:10.1371/journal.pone.0225163)
Supplement: S1 Table — (DOCX) [file pone.0225163.s001.docx]

**S1 Table. Baseline demographics by inclusion and exclusion**

| \|  \| 3 or more data points available \| 2 or fewer data points available \| \| --- \| --- \| --- \| \|  \| (N = 960) \| (N = 194) \| |
| --- | --- | --- | --- | --- | --- | --- |
| \| **Maternal Age (years)** \|  \|  \| \| --- \| --- \| --- \| \| Mean (SD) \| 26.70 (4.48) \| 26.76 (4.86) \| \| Median (Q1, Q3) \| 26.0 (24.0, 30.0) \| 27.0 (23.0, 30.0) \| \| % Missing (Min, Max) \| 0.0% (18.0, 45.0) \| 0.0% (18.0, 41.0) \| \| **SES Asset Index Score** \|  \|  \| \| Mean (SD) \| 0.03 (1.61) \| -0.17 (1.70) \| \| Median (Q1, Q3) \| 0.4 (-0.9, 1.2) \| 0.1 (-1.2, 1.1) \| \| % Missing (Min, Max) \| 0.0% (-5.0, 2.8) \| 0.0% (-4.5, 2.5) \| \| **Maternal Education (years)** \|  \|  \| \| None (0) \| 135 (14.1%) \| 35 (18.0%) \| \| Primary (1-5) \| 191 (19.9%) \| 35 (18.0%) \| \| Middle (6-8) \| 180 (18.8%) \| 35 (18.0%) \| \| Secondary (9-10) \| 245 (25.5%) \| 48 (24.7%) \| \| Higher Secondary (11-12) \| 94 (9.8%) \| 15 (7.7%) \| \| Tertiary (>12) \| 115 (12.0%) \| 26 (13.4%) \| \| **Number of living children** \|  \|  \| \| First pregnancy \| 280 (29.2%) \| 69 (35.6%) \| \| 1 to 3 \| 596 (62.1%) \| 103 (53.1%) \| \| 4 \| 84 (8.8%) \| 22 (11.3%) \| \| **Household structure** \|  \|  \| \| Nuclear \| 208 (21.7%) \| 52 (26.8%) \| \| Joint/extended \| 636 (66.3%) \| 123 (63.4%) \| \| Multiple households \| 116 (12.1%) \| 19 (9.8%) \| \| **Long standing illness, disability or infirmity?** \|  \|  \| \| No \| 821 (85.5%) \| 170 (87.6%) \| \| Yes \| 139 (14.5%) \| 24 (12.4%) \| |

SES: Socioeconomic Status
