# Supplementary material for: Psychosocial determinants of sustained maternal functional impairment: Longitudinal findings from a pregnancy-birth cohort study in rural Pakistan
Source: PLoS One. 2019 Nov 19;14(11):e0225163. doi: 10.1371/journal.pone.0225163 (PMC6863521; doi:10.1371/journal.pone.0225163)
Supplement: S2 Table — (DOCX) [file pone.0225163.s002.docx]

**S2 Table. Baseline demographics by all time points available versus three time points**

| \|  \| All four data points available \| 3 data points available \| \| --- \| --- \| --- \| \|  \| (N = 783) \| (N = 177) \| |
| --- | --- | --- | --- | --- | --- | --- |
| \| **Age (in years).** \|  \|  \| \| --- \| --- \| --- \| \| Mean (SD) \| 26.65 (4.36) \| 26.89 (4.97) \| \| Median (Q1, Q3) \| 26.0 (24.0, 30.0) \| 27.0 (24.0, 30.0) \| \| % Missing (Min, Max) \| 0.0% (18.0, 40.0) \| 0.0% (18.0, 45.0) \| \| **SES Asset Index Score** \|  \|  \| \| Mean (SD) \| 0.05 (1.60) \| -0.06 (1.67) \| \| Median (Q1, Q3) \| 0.4 (-0.8, 1.2) \| 0.3 (-1.1, 1.1) \| \| % Missing (Min, Max) \| 0.0% (-5.0, 2.8) \| 0.0% (-4.7, 2.8) \| \| **Maternal Education (years)** \|  \|  \| \| None (0) \| 107 (13.7%) \| 28 (15.8%) \| \| Primary (1-5) \| 151 (19.3%) \| 40 (22.6%) \| \| Middle (6-8) \| 150 (19.2%) \| 30 (16.9%) \| \| Secondary or more (6-12+) \| 375 (47.9%) \| 30 (16.9%) \| \| **How many living children do you have?** \|  \|  \| \| First pregnancy \| 224 (28.6%) \| 56 (31.6%) \| \| 1 to 3 \| 494 (63.1%) \| 102 (57.6%) \| \| 4 \| 65 (8.3%) \| 19 (10.7%) \| \| **Household structure** \|  \|  \| \| Nuclear \| 171 (21.8%) \| 37 (20.9%) \| \| Joint/Multiple households \| 612 (78.2%) \| 140 (79.1%) \| \| **Long standing illness, disability or infirmity?** \|  \|  \| \| No \| 663 (84.7%) \| 156 (88.1%) \| \| Yes \| 120 (15.4%) \| 21 (11.9%) \| |

SES: Socioeconomic Status
