# Supplementary material for: Psychosocial determinants of sustained maternal functional impairment: Longitudinal findings from a pregnancy-birth cohort study in rural Pakistan
Source: PLoS One. 2019 Nov 19;14(11):e0225163. doi: 10.1371/journal.pone.0225163 (PMC6863521; doi:10.1371/journal.pone.0225163)
Supplement: S3 Table — (DOCX) [file pone.0225163.s003.docx]

**S3 Table. Predictors of group trajectory membership (reference group 1, persistent high functioning (n=509))**

|  | Unadjusted bivariate comparisons | | | |
| --- | --- | --- | --- | --- |
|  | Moderate Recovery, Group 2 (n=366) | | Chronic poor functioning, Group 3 (n=85) | |
|  | OR | CI | OR | CI |
| **Demographic and Socioeconomic** |  |  |  |  |
| Maternal Age at baseline | 1.01 | 0.96-1.05 | 1 | 0.93-1.07 |
| Number of Living Children (nulliparous is comparison) |  |  |  |  |
| 1 to 3 | 1.29 | 0.90-1.82 | 1.43 | 0.65-3.12 |
| 4 or more | 1.69** | 1.21-2.34 | 2.3** | 1.25-4.21 |
| SES Asset Index Score | 0.68*** | 0.57-0.82 | 0.5*** | 0.40-0.63 |
| Maternal Education (none is comparison) |  |  |  |  |
| Primary (1-5) | 0.67 | 0.42-1.07 | 0.92 | 0.54-1.57 |
| Middle (6-8) | 0.54* | 0.33-0.87 | 0.36* | 0.16-0.80 |
| Secondary or more (9-12+) | 0.45*** | 0.28-0.71 | 0.17*** | 0.07-0.37 |
| Household structure (joint is comparison) |  |  |  |  |
| Multiple households | 1.06 | 0.67-1.70 | 2.69** | 1.47-4.92 |
| Nuclear household | 1.73** | 1.17-2.55 | 2.85** | 1.51-5.39 |
| **Illness** |  |  |  |  |
| Chronic illness or disability at baseline | 2.25** | 1.33-3.82 | 2.93*** | 1.65-5.20 |
| Depression (PHQ-9) | 1.15*** | 1.11-1.18 | 1.31*** | 1.24-1.39 |
| **Psychosocial Factors** |  |  |  |  |
| Social Support (MSPSS) | 0.67*** | 0.59-0.76 | 0.48*** | 0.40-0.59 |
| Life events | 1.27*** | 1.18-1.36 | 1.57*** | 1.42-1.73 |
| Perceived Stress (PSS) | 1.1*** | 1.08-1.12 | 1.2*** | 1.15-1.24 |
| Sampling weights applied in all models. SES=socioeconomic status | | | | |
| *p-value <0.05; ** p-value <0.01; ***p-value<0.001 | | |  |  |
